# The Importance of CD44 as a Stem Cell Biomarker and Therapeutic Target in Cancer

**DOI:** 10.1155/2016/2087204

**Published:** 2016-04-21

**Authors:** Ranjeeta Thapa, George D. Wilson

**Affiliations:** ^1^Department of Radiation Oncology, Beaumont Health System, Royal Oak, MI 48073, USA; ^2^Department of Physics, Oakland University, Rochester, MI 48309, USA

## Abstract

CD44 is a cell surface HA-binding glycoprotein that is overexpressed to some extent by almost all tumors of epithelial origin and plays an important role in tumor initiation and metastasis. CD44 is a compelling marker for cancer stem cells of many solid malignancies. In addition, interaction of HA and CD44 promotes EGFR-mediated pathways, consequently leading to tumor cell growth, tumor cell migration, and chemotherapy resistance in solid cancers. Accumulating evidence indicates that major HA-CD44 signaling pathways involve a specific variant of CD44 isoforms; however, the particular variant almost certainly depends on the type of tumor cell and the stage of the cancer progression. Research to date suggests use of monoclonal antibodies against different CD44 variant isoforms and targeted inhibition of HA/CD44-mediated signaling combined with conventional radio/chemotherapy may be the most favorable therapeutic strategy for future treatments of advanced stage malignancies. Thus, this paper briefly focuses on the association of the major CD44 variant isoforms in cancer progression, the role of HA-CD44 interaction in oncogenic pathways, and strategies to target CD44-overexpressed tumor cells.

## 1. Background

In cancer biology, cancer cell progression is defined by increased proliferation, invasion, migration, and metastasis of cancerous cells to other parts of the body. Tumor cell heterogeneity plays a major role in cancer progression and metastasis [1]. This heterogeneity was initially attributed to clonal expansion, in which various clones are frequently generated due to the sequential genetic and/or epigenetic alterations in response to certain carcinogens during cancer development, with the daughter cells of more dominant clones overtaking the cells of other malignant clones in a wave-like fashion. However, an alternative view also exists called the cancer stem cell (CSC) hypothesis. According to this hypothesis, heterogeneity and hierarchy among all of the cells exist as a consequence of asymmetric division of cancer stem cells (CSCs) within the tumor mass, and all other cells comprising the tumor bulk are the result of differentiated CSCs [2]. The CSCs have ability to self-renew and form pools of precursors like normal stem cells; however, CSCs demonstrate deregulated self-renewal/differentiation processes and generate daughter cells that are arrested at various stages of differentiation [3].

**Figure 1:**
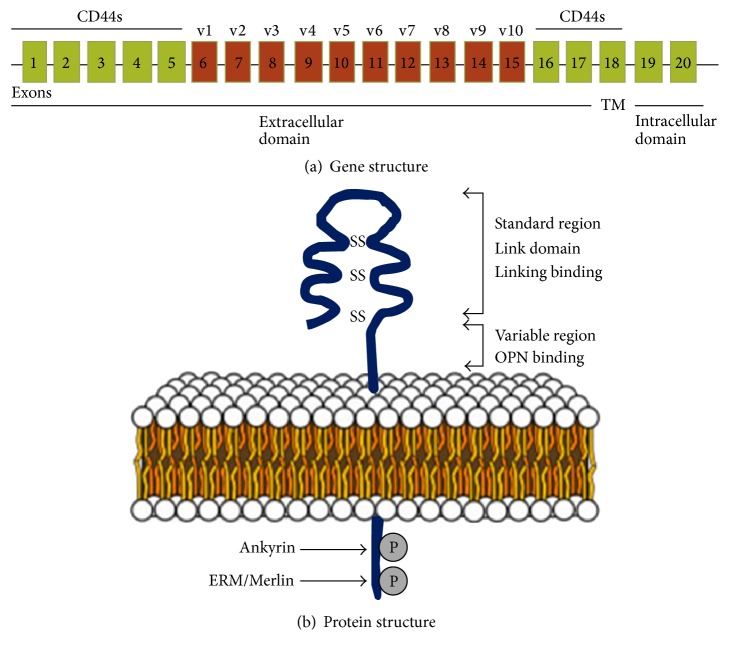
The plot shows (a) CD44 gene and (b) protein structure. Figure is adapted from Louderbough and Schroeder, 2011 [161].

Many studies support the role of CSCs and their specific markers associated with the malignancies. One of the compelling markers in tumor malignancies is cluster of differentiation 44 (CD44). The CD44 antigen is a single polypeptide chain, single pass, and cell surface glycoprotein encoded by the CD44 gene [4]. CD44 is a large highly conserved and complex gene, which consists of 19 exons located on human chromosome 11 and mouse chromosome 2 [5, 6]. In the human CD44 gene, exons 1–5 and 16–20 produce the standard form of CD44 (CD44s; ~85 kDa). The remaining exons 6–15 are alternatively spliced to form the variant forms of CD44 (CD44v) and referred to as variant exons 1–10 (v1–10) [7, 8] (Figure 1). Ten CD44v exons are detected in the mouse, and nine variant exons are detected in man. Alternative splicing and posttranslation modification are highly regulated in CD44v isoforms and, theoretically, multiple splicing possibilities could give rise to many alternative varieties of CD44v isoform. However, very few of them have been verified experimentally [9, 10].

Several experimentally verified CD44v forms have been shown to be directly involved in many malignant tumors and some correlate with metastatic progression [10] (Table 1). CD44v isoforms are differentially expressed in both normal and malignant cells, and the existence of CD44 isoform expression is clearly confirmed by both histological and cellular studies [11]. Table 1 describes the major CD44v associated with cancer progression and metastasis.

Recent evidence has strengthened the potential role of CD44 in CSCs and their influence on disease progression and treatment outcome. In solid cancers CSCs were first defined based on CD44 expression by flow cytometry as CD44^bright^ and CD44^dim^ populations. It has been shown that CD44^bright^ but not CD44^dim^ is capable of regenerating a heterogeneous tumor and demonstrating self-regeneration when transplanted into immune-deficient mice [12]. CD44^bright^ also expresses high levels of the stem cell marker BMI-1 gene, encoding a self-renewal protein found in embryonic stem cells that costains with cytokeratin 5/14, a basal cell marker. This transmembrane protein is commonly associated with many physiological and pathological processes when it is bonded to certain ligands [9].

Although other extracellular matrix components such as osteopontin, collagens, growth factors, and metalloproteinases can bind to CD44, hyaluronan (HA) is the most common and immediate ligand for CD44. All isoforms of the CD44 variants membrane receptor share a common ligand-binding region for HA [11, 13]. HA is produced by hyaluronan synthase, an integral plasma membrane protein, and is released directly into the extracellular matrix. HA is a large, linear, and anionic polysaccharide which is composed of tandem disaccharide repeats of b-1,4-D-glucuronic acid-b-1,3-D-N-acetylglucosamine [14, 15]. As an important component of the extracellular matrix, HA contributes significantly in many cellular processes, for example, cell adhesion, cell migration, innate immunity, wound healing, and cancer progression [16].

**Figure 2:**
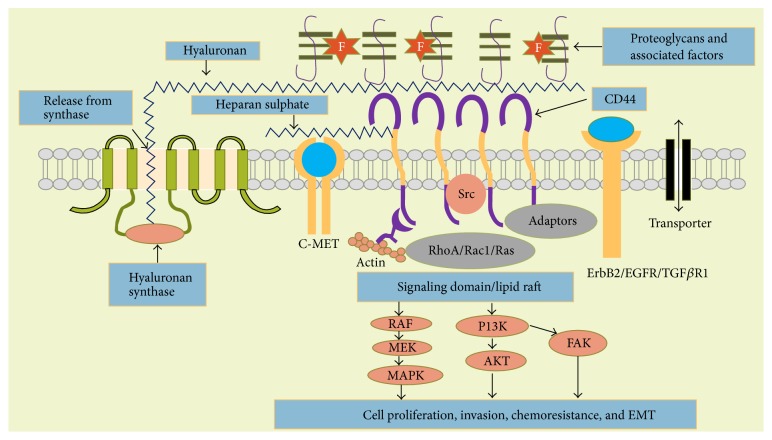
The influence of tumor cell produced by hyaluronan on cell signaling cascades. Figure is adapted from Toole, 2009 [78].

An accumulation of evidence indicates that HA-CD44 interaction in the extracellular domain promotes multiple signaling pathways which play a crucial role in tumor cell progression in a variety of solid tumor malignancies (Figure 2). As described in Figure 2, HA is produced and extruded by hyaluronan synthases in the plasma membrane and is directly released into the extracellular matrix. This HA interacts multivalently with CD44 to activate/regulate many signaling domains within the plasma membrane such as receptor tyrosine kinases (ErbB2 and EGFR) and transforming growth factor-*β* receptor type 1 (TGF*β*R1) [17]. In addition, the HA-CD44 interaction also mediates nonreceptor kinases (Src family) or Ras family GTPases [18]. The HA-CD44 interaction further facilitates the complex formation of several adapter proteins such as Vav2, Grb2, and Gab-1 which mediate the interaction of CD44 with upstream effectors like RhoA, rac1, and Ras [18–20]. These receptors further activate several oncogenic pathways such as the mitogen activated protein kinases (MAPK) and PI3 kinases/akt pathways that consequently promote tumor cell proliferation, survival, migration, invasion, and chemoresistance [18]. In some cases, heparan sulphate chains (carbohydrate side groups on CD44 variant region) associate with regulatory growth factors that activate C-Met receptors which further drive these oncogenic pathways [21]. In addition, HA-CD44 interaction also stimulates multidrug and metabolic transporters that are strongly associated with therapy resistance [17, 19, 22]. Finally, HA-CD44 interaction induces cytoskeletal changes that promote tumor cell motility and invasion [21, 23, 24]. The bulk of the current evidence suggests that different CD44 variants are associated with these interactions [11, 25–31].

## 2. Different CD44v Association with Cancer Progression

A single CD44 polypeptide is divided into three major domains: extracellular binding domain, transmembrane domain, and cytoplasmic domain. Further, all CD44v isoforms contain a conserved extracellular binding domain for HA and a common cytoplasmic domain for triggering cell signaling pathways. The coupling of HA with the CD44 extracellular domain correlates with a multiple signaling kinases transduction in the cytoplasmic domain, which determines how one family of molecules regulates several cellular processes. CD44v isoforms are expressed in both normal and tumor cells at different levels, indicating that the CD44 isoforms are also an essential component for normal cellular functions [32]. As mentioned earlier, exon splicing mechanisms can lead to the overexpression of CD44v isoforms in cancer cells; their role and the degree of expression vary in different malignancies. In certain cancers, CD44v isoforms are considered to be tumor progression promoters [33–38], while in other cancers, they may be involved as tumor suppressors [26, 39–44].

There are many possible factors for these varying results. For example, different research groups use different methods for detecting CD44 such as immunohistochemistry or PCR with different antibodies which makes it difficult to compare the results since some CD44v epitopes may not be targeted by some of the antibodies due to the posttranslational modification, resulting in conformational changes of the protein. In addition, tumor heterogeneity may also play a major role in these discrepant results [45]. This section only focuses on the role of CD44v as tumor promoters in some major cancers.

### 2.1. Colorectal Cancer

In colon cancer, CD44v3 has been shown to activate invasion and resistance to apoptosis, and CD44v6 has been associated with tumor metastasis and decreased disease-free survival [30, 46]. Yamaguchi et al. [47] showed that CD44v8–10 isoforms play a role in metastasis of colorectal cancer and are useful independent factors for the prediction of prognosis in colorectal cancer patients.

Further, Du et al. [25] demonstrated that a single CD44^bright^ cell from a tumor could form a sphere* in vitro* which had characteristic stem cell properties and was able to generate a xenograft tumor resembling the properties of the primary tumor. Also, knockdown of CD44 strongly prevented clonal formation and inhibited tumorigenicity in a xenograft model, concluding that CD44 had a potential to be a CSC marker for colorectal cancer (CRC). In addition, Ozawa et al. [48] evaluated primary CRC cell isolates to determine the significance of several CSC markers, including CD44, as predictors of tumorigenesis and prognosis. CD44-positive cells from fresh clinical samples of CRC were differentiated by flow cytometric sorting and evaluated for tumorigenicity following subcutaneous transplantation into NOD/SCID mice. Cancer stem cell marker expression was tested in both xenografts and a complementary DNA library compiled from CRC patient samples. They demonstrated that CD44^bright^ populations were significantly more tumorigenic than the total cell population. The clinical samples expressed several CD44 variants with CD44v2 being specifically overexpressed in both primary tumors and xenografts in comparison with the normal mucosa. A prognostic assay using qRT-PCR showed that the CD44v2^bright^ group had a significantly worse prognosis compared to the CD44v2^dim^ group, concluding the prognostic significance of CD44v2 upregulation in CRC. Recently Todaro et al. [49] showed that colorectal cancer stem cells (CR-CSCs) express CD44v6, which is both a functional biomarker and therapeutic target and is essential for their migration and generation of metastatic tumors.

### 2.2. Lung Cancer

In a study with squamous cell carcinoma and bronchioalveolar carcinoma of lung malignancies, CD44v5 and CD44v6 have been shown to promote tumor metastasis [29, 50]. Miyoshi et al. [51] showed that CD44v3, CD44v5, CD44v6, or CD44v7 was expressed in 28 of 31 (90.3%) non-small cell lung carcinomas (NSCLCs) tissue samples using RT-PCR, and the expression of the CD44v6 was associated with lymph node metastasis. Recently, it has been shown that CD44v6 expression in NSCLC is associated with squamous subtype, lymph node metastasis, and a poor survival and should be considered as a new important prognostic marker [52–54].

Further, Leung et al. [55] investigated whether the stem cell hypothesis was applicable to lung cancers by using ten lung cancer cell lines (H1650, HKULC2, H1299, HKULC4, HCC827, H23, HCC1833, A549, H441, and H1648). They screened the expression profile of CD44 along with two other putative surface markers CD34 and CD133 and nuclear markers BMI1 and OCT4 by flow cytometry. There was variation in the expression level of all the surface markers tested, and CD44 was the major marker expressed by H1299 and H23 cells. Both the nuclear markers, BMI1 and OCT4, were expressed in the majority of cancer cells in all cell lines studied. Further, CD44^bright^ cells of four cell lines showed spheroid body formation and* in vivo* tumor initiation ability. When CD44^bright^ cells of H1299 cell line were used for the testing of* in vivo* tumor transplantability, the primary xenografts consisted of mixed CD44^bright^ and CD44^low^ cells in similar ratios as the parental H1299 cell line, supporting* in vivo* differentiation. Using RT-PCR study, they showed that both freshly sorted CD44^bright^ and CD44^low^ cells derived from CD44^bright^-initiated tumors expressed the pluripotency genes OCT4/POU5F1, NANOG, and SOX2 (stemness markers); however CD44^low^ did not. Further, CD44^bright^ cells were more cisplatin resistant than CD44^low^ cells, concluding that stem cell-like properties are enriched in CD44 expressing subpopulations of some lung cancer cell lines.

### 2.3. Breast Cancer

In breast cancer, CD44v3, CD44v5, and CD44v6 have been associated with metastasis [27]. Tempfer et al. [56] investigated the expression of CD44 isoforms CD44v5, CD44v6, and CD44v7-8 in 115 human breast cancer specimens by means of immunohistochemistry and found that these variants are strongly associated with axillary lymph node metastasis. Ryś et al. [57] estimated the frequency of CD44 expression as well as two CD44 isoforms CD44v3 and CD44v5 in female breast cancer, concluding that CD44v3 significantly correlated with the presence of metastases to the lymph nodes.

Further, CD44^bright^/CD44^+^ cells either singly or in combination with other stem cell markers have shown tumorigenic potential [28]. In a study with breast cancer cells (MDA-MB-231, MDA-MB-436, Hs578T, SUM1315, and HBL-100 cell lines) having CD44^+^/CD24^−^ subpopulation showed higher levels of expression of proinvasive genes and had highly invasive properties [58]. AL-Hajj et al. [59] initially identified and isolated the tumorigenic cells as CD44^+^CD24^−/low^ lineage^−^ in breast cancer tissues from eight of nine patients. As few as 100 cells with this phenotype were able to form tumors in mice, while tens of thousands of cells with alternate phenotypes were unable to form tumors. The tumorigenic subpopulation could be serially passaged, and the tumor formed in each time of passage contained mixed population of additional CD44^+^CD24^−/low^ lineage^−^ tumorigenic cells as well as the phenotypically diverse mixed populations of nontumorigenic cells present in the original tumor.

Further, Olsson et al. [60] demonstrated that CD44 variants were heterogeneously expressed in breast cancer and correlated with tumor subtypes and cancer stem cell markers. They showed that a high expression of CD44v2–10 isoform, which retain all variant exons, was correlated to positive steroid receptor status, low proliferation, and luminal A subtype. The CD44v3–10 showed similar correlation, while high expression of CD44v8–10 was correlated to positive EGFR, negative/low HER2 status, and basal-like subtype. Further, the CD44 variants described above were associated with all tumors that were characterized as positive for CD44^+^/CD24^−^ phenotype by immunohistochemistry. These findings suggested the involvement of CD44 variants in specific oncogenic signaling pathways.

### 2.4. Leukemia

In leukemia, several CD44 variants are overexpressed in malignant hematopoietic cells and strongly involved in metastasis and shorter survival rates [31]. For example, bone marrow progenitors express CD44v3, CD44v6, CD44v9, and CD44v10, while lymphocytes and monocytes express CD44v3, CD44v6, and CD44v9 following stimulation with inflammatory cytokines [61]. CD44 variant expression has been associated with poor prognosis and increased metastatic spread in a number of hematological and nonhematological malignancies. Overexpression of CD44v6, CD44v9, and CD44v10 has been associated with poor prognosis in non-Hodgkin's lymphoma and myeloma and CD44v6 with poor prognosis and shorter survival rate in acute myeloid leukemia (AML) [31]. In acute lymphoblastic leukemia (ALL), CD44v6 was shown to be expressed on bone marrow cells from patients with poor risk ALL, but not on those from patients with a good prognosis [62, 63].

Permanent cure of leukemia requires elimination of leukemic stem cells (LSCs), the only cell type capable of initiating and maintaining the leukemic clonal hierarchy. For example, targeting of the stem cell marker CD44, highly expressed in AML cells, resulted in eradication of leukemic stem cells (LSCs) [64].

Though blockade of CD44 is considered a therapeutic option for the elimination of LSCs, anti-panCD44 can interfere with hematopoiesis. Thus, targeting CD44 variant isoforms in leukemic malignancies can inhibit leukemic growth without attacking hematopoiesis. For example, recently, Erb et al. [65] showed that CD44s and CD44v10 expression distinctly influenced niche embedding of hematopoietic stem cells and targeting CD44v10 with anti-CD44v10 prolonged the survival time.

### 2.5. Pancreatic Cancer

In pancreatic cancer, different CD44v isoforms are associated with malignancies. Metastasis-specific isoforms of CD44 were first documented in a model of rat pancreatic adenocarcinoma (CD44v4–7 and CD44v6-7) and subsequently in other cancers [41]. Rall and Rustgi [41] also investigated that CD44v6 isoform or CD44v8–10 are expressed in primary and metastatic human pancreatic adenocarcinoma, which is usually metastatic at the time of diagnosis and has the worst prognosis of all gastrointestinal cancers [41, 66]. They used radiolabeled RT-PCR/PAGE and Southern blot hybridization to analyze clinical specimens of primary and metastatic pancreatic cancer for the expression of CD44s, CD44v8–10, and CD44v6 isoforms. There was no difference in the expression of CD44s and CD44v8–10 among the primary and metastatic adenocarcinomas and the control specimens of pancreata. However, CD44v6 was found in metastatic lesions. Later, Gansauge et al. [67] showed that CD44v6 were expressed on both adenocarcinoma and normal pancreatic cells, while CD44v5 were strongly expressed on adenocarcinomas. Gotoda et al. [68] further tested whether CD44v6 is a useful marker for evaluating the prognosis of pancreatic cancer patients. They also attempted to assess the clinicopathological implications of the CD44v2 for pancreatic cancer. Their results showed that both CD44v6 and CD44v2 were expressed on tumor cells, and their expression was correlated with decreased overall survival.

Recently, Li et al. [36] studied the expression pattern of CD44v2–CD44v10 and CD44s and found that high expression of CD44v6 and CD44v9 and low expression of CD44s (CD44v6^+^, CD44v9^+^, and CD44s^−^) were associated with pancreatic carcinoma metastasis and progression and that CD44v6^+^/CD44s^−^ was an independent risk factor affecting overall survival.

Further, Kiuchi et al. [69] demonstrated that pancreatic cancer cells (PCCs) with an epithelial phenotype upregulate cell surface expression of CD44v9, an important CSC marker, during the mitotic phases of the cell cycle. They found PCCs with an epithelial phenotype upregulated cell surface expression of CD44v9 in prophase, metaphase, anaphase, and telophase and downregulated CD44v9 expression in late-telophase, cytokinesis, and interphase. In addition, flow-sorted CD44v9-negative PCI-55 cells resumed CD44v9 expression when they reentered the mitotic stage and CD44v9 (bright) mitotic cells showed intracellular expression of the multidrug resistance protein 1 (MDR1).

### 2.6. Head and Neck Cancer

In head and neck cancer, Reategui et al. [70] first characterized the expression of CD44v3-containing isoforms. Both cell culture and histological studies were performed using HNSCCs cell lines and tissues. The tissue study identified that high levels of CD44v3 were expressed in tumor tissues compared to normal tissue [70]. The cellular study showed that an increased level of CD44v3 did not affect the rate of proliferation; however, a significant increase in migration was observed [70]. Wang et al. [11, 13] reported studies utilizing HNSCC cell lines and clinical tissue specimens and found that CD44v3, CD44v6, and CD44v10 isoforms were associated with HNSCC lymph node metastasis and advanced T status, perineural invasion and decreased survival, and distant metastasis and radiation failure, respectively. Kawano et al. [71] used immunohistochemical analysis using monoclonal antibody against CD44v6 isoforms in paraffin-embedded mesopharyngeal cancer tissues and found that CD44v6 expression correlated with tumor invasion, lymph node metastasis, and shorter survival.

Recently CD44v9 has emerged as a novel marker of cancer stemness in a variety of solid tumors including HNSCC [72–77]. The CD44v9 is active though increasing the intracellular levels of glutathione (GSH) when combined with the functional subunit of the cystine/glutamate transporter (xCT), resulting in cellular protection from reactive oxygen species (ROS) and oxidative stress, which is one of the distinct characteristics of CSCs [72, 73].

Aso et al. [73] evaluated the expression levels of CD44v9 protein in clinical samples (biopsy and surgically removed tumor specimens) of 102 patients following induction concurrent chemoradiotherapy (CCRT). They found that CD44v9 expression level in biopsy specimens did not correlate with the patients having favorable response to CCRT or their survival. However, in nonresponding patients, the CD449-positive group showed significantly worse prognosis compared to the CD44v9-negative group. Based on tumor (T), node (N), response to CCRT, and CD44v9 positivity analyses, the CD44v9 positivity was significantly correlated with poor prognosis, along with advanced N stage. Further, the survival rate of the CD44v9-induced group was significantly worse compared to the CD44v9-noninduced group. These results concluded that CCRT-induced CD44v9-expressing CSCs appear to be a major hurdle to CCRT.

## 3. Use of Anti-CD44 Monoclonal Antibodies to Target CD44

Targeting CD44 using monoclonal antibody-mediated pathways is a novel targeted therapy in cancer treatment. Anti CD44 antibodies developed against various highly expressed CD44v variants have the potential to inhibit and disrupt CD44-matrix interactions. There are two basic strategies in which either the native antibody is employed to bind and neutralize the receptor by competitive inhibition of its ligand consequently preventing the receptor-signaling cascade or radioisotopes, toxins, or chemotherapeutic agents can be attached to the antibodies to cause cancer cell death. More recently, the use of antibody-attached nanoparticle systems is receiving increasing attention.

A study with human acute myeloid leukemia (AML) cell showed that an activated anti-CD44 antibody (H90) reduced the leukemic repopulation by alteration of the behavior of AML leucocyte stem cells (LSC) by annulling AML LSC homing, resulting in tumor-initiating cell death [64]. Greater survival rates were also observed in mice xenografts of BCR-ABL-expressing leukemic cells after blockade of CD44 by anti-CD44 antibodies [78, 79]. Verel and colleagues developed a chimeric (BIWA-2) and two humanized (BIWA-4 and BIWA-8) monoclonal antibodies against CD44v6 which were derivatives of the BIWA-1 monoclonal antibody [80] to target CD44v6 in head and neck cancer xenografts. In comparison with the murine monoclonal antibody, U36, and BIWA-1, they showed that the MAbs bound to CD44v6 with an up to 46-fold difference in affinity ranking: U36 < BIWA-4 < BIWA-8 < BIWA-1 ~ BIWA-2. In terms of biodistribution* in vivo*, significant differences were observed between the pairs: U36 versus BIWA-1 (35.0-fold difference), BIWA-4 versus BIWA-2 (14.0-fold), and BIWA-4 versus BIWA-8 (4.0-fold). When the antibodies were assessed and radioimmunotherapeutics (RIT) labeled with ^186^Re, the lower-affinity monoclonal antibodies (such as U36 and BIWA-4) showed a higher degree and specificity of tumor localization. In other studies, BIWA-4 was radiolabeled with Tc-99 and Re-186 and conjugated with a cytotoxic drug, mertansine, and tested for its efficacy in targeting CD44v6 antigen [81, 82]. These studies verified that radiolabeled BIWA-4 can be administered safely and showed some promising results in clinical trials with HNSCC patients without any human anti-human antibody (HAHA) responses. However, a phase I dose escalation study with HNSCC patients exhibited dose limiting skin toxicity in nontumor surrounding tissue likely due to CD44v6 expression in normal keratinocytes [83]. The majority of skin toxicity was reversible; however, the incident led to the discontinuation of the study with the conclusion that this antibody was not suitable for human studies. A similar study in the following year was performed to target CD44v6 with the prodrug bivatuzumab mertansine (BIWI 1) and deconjugated BIWI 1 in a dose escalation phase I clinical trial with 31 HNSCC patients [84]. The purpose of this study was to characterize the pharmacokinetics and immunogenicity and safety of these immunoconjugates. Both agents were administered safely and found to be appropriate as novel conjugates with the maximum tolerated dose (300 mg/m^2^) of this novel approach. These studies suggest CD44, particularly CD44v6 isoform, remains an attractive target for cancer treatments.

In alternative approaches nanoprobes, such as the combination of nanorods and tumor sensitizing drugs, have been investigated [85]. Anti-CD44 antibodies-conjugated gold nanorods have been used to sensitize MCF-7 breast cancer that overexpresses the CD44 surface marker [85]. The absorption of near infrared light by the gold nanorod led to a local rise in the temperature; as a result, photoablation of the CD44^bright^ cells occurred.

## 4. Use of HA Oligomers

The disruption of HA-CD44 interaction by using HA oligomers is another approach to target CD44. This approach comprises replacing the multivalent interaction of high molecular weight (HMW) HA and CD44 with monovalent interaction of small oligomers of HA (6–18 saccharide units of HA).

HA is a widely known HMW glycosaminoglycan polymer from which oligosaccharides of desired size can be readily obtained [86]. HA is produced by cell membrane-bound protein called hyaluronan synthase (HAS) [87]. There are three types of HASs involved in HA biosynthesis: HAS-1, HAS-2, and HAS-3 [88]. HAS-1 is encoded by the gene* has1* linked on 19q13.3 human chromosome. HAS-2 is encoded by the* has2* gene localized at chromosome 8q24.12. It is responsible for generation of HA in response to shock, inflammation, and tissue repair. HAS-3 is encoded by* has3* gene localized on chromosome 16q22.1 [87, 89]. HAS-1 and HAS-3 generate HA with broad size distributions (200,000 to two million Daltons), whereas HAS-3 generates HA with extremely large sizes (>two million Daltons) [90–92].

Small oligomers of HA suppress antiapoptotic signaling pathways in cancer cells and inhibit the activity of transporters that enhance the multidrug resistances to some chemotherapeutic agents [17, 93]. Initial studies showed that HA oligomers of 3–9 disaccharides bind CD44 monovalently and displace stromal HA polymer bound to membrane receptor [94, 95]. Recently these oligomers have also been shown to inhibit HA synthesis [96]. HA oligomer treated tumor cells show disassembly of CD44-transporter and receptor tyrosine kinase (TRK) complexes, internalization of these disassembled components, and weakening of their function [96, 97].* In vivo* treatments which inject small HA oligomers, but not the large polymers, induce tumor regression in many human xenograft experiments with various cancer types such as melanoma, carcinoma, glioma, osteosarcoma, and malignant peripheral nerve sheath tumors [98]. Ween et al. [99] reported that small HA oligomers (6–10 disaccharides) were able to block cancer cell adhesion, motility, and invasion in both presence and absence of exogenous HA. In this study, they used three different ovarian cancer cells (OVCAR-3, OVCAR-5, and SKOV-3) and artificially induced tumor cell motility, invasion, and metastasis in these cells by adding versican and/or exogenous HA, and then, they were treated with HA oligomers. They concluded that HA oligomers are promising inhibitors of ovarian cancer dissemination. In another study, Zeng et al. [86] showed that injecting HA oligomer can potentially inhibit* in vivo* tumor formation using B16F10 melanoma cell lines. Urakawa et al. [100] studied the effective size of the HA-oligosaccharides required to inhibit the cell growth in highly invasive breast cancer cell line, MDA-MB-231 by testing the effects of HA tetrasaccharides, HA decasaccharides, and high molecular weight HA. The results showed that HA decasaccharides significantly inhibited cell growth, motility, and invasion, whereas HA tetrasaccharides could not. Further, HA disaccharides inhibited the expansion of osteolytic lesions in a mouse bone metastasis model of breast cancer. From this study, they concluded that “HA-oligosaccharides suppressed progression of bone metastasis in breast cancer via interruption of endogenous HA-CD44 interaction and, as such, could be a novel therapeutic candidate to limit bone metastasis of breast cancer.” Moreover, small HA oligomers have shown their potential during* in vivo* treatments by suppressing tumor growth and/or inducing tumor regression in experiments using xenografts of several tumor types [17, 86, 96, 98, 101, 102].

## 5. Use of Hayaluronidases

HA can be catabolized by enzymatic and nonenzymatic processes. In the enzymatic process, hyaluronidases (HAases or HYALs) are a class of enzymes that predominantly degrade HA, though they have limited ability to degrade chondroitin and chondroitin sulphates [78, 103]. Human HYALs are encoded by six genes:* hyall1, hyal2,* and* hyal3* localized at 3p21.3 human chromosome and* hyal4, ph-20* (or* spam1*), and pseudogene* phyal1* (that lost its protein-coding ability) localized at chromosome 7q31.3 [88]. Regarding the role of HYALs in cancer, a considerable body of data exist supporting HYALs overexpression and elevated activity in many cancers [104, 105]. Clinical data demonstrate that both* hyal1* and* hyal2* genes are overexpressed in advanced stages of colorectal disease [104].* In vitro* knockdown of* hyal1* gene expression in breast cancer cells (MCF7 and ZR-75-30 cells) showed reduced cell growth, adhesion, invasion, and angiogenesis, while induced overexpression of the isoenzyme elevated cell malignancy. Further,* in vivo* study using MCF7-cells demonstrated that induced* hyal1* overexpression in a nude mouse model resulted in increased tumor growth and promoted angiogenesis [105]. A variety of* hyal1-*expressing tumors such as bladder and prostate, genitourinary tract, head and neck, and brain show a significant amount of expansion in micro vessel density and larger capillaries compared to the non-*hyal1-*expressing tumors [106–109]. Due to their overexpression in some types of malignancies, HYALs have been considered as a diagnostic marker of the disease [110, 111].

Despite the fact that constitutive HYALs may promote prooncogenic activity of HA, overexpression or exogenous administration of excess amounts of HYALs inhibits tumor potential [103, 106, 107]. Experimental and clinical results of HYALs showed that they can be used as an adjunct to chemotherapy by improving the access of the drugs to the cancer cells which is attributed to their properties of accelerating the transport of numerous endogenous and exogenous substances within the tissue by loosening the cell-cell contact and the intercellular connective matrix [106, 112]. Shuster et al. [113] demonstrated that the tumor volumes in human breast cancer xenografts were significantly decreased by up to 50% upon the intravenous administration of an extremely high dose of HYALs. HYALs have shown their potential to sensitize mouse mammary carcinoma cells (EMT-6 cells) which are sensitive to their antiadhesive effects [78, 114]. Generally HYALs are active by reducing drug diffusion barriers; however, they may also work through degrading high molecular weight HA into the low molecular weight HA [17].

These opposing data described above suggest that HYALs may act as both a tumor promoter and tumor suppressor [78, 106]. To resolve this paradox about their role in cancer, further investigations have been performed [78, 106]. For example, it has been shown that HYAL-1 acts as a tumor promoter at a naturally expressed level by tumor cells, while above the naturally expressed level (exceeding 100 mU/10^6^ cells) it acts as tumor suppressor through inducing apoptosis [78, 106]. Thus, the function of HYALs (which are not tumor cell-derived), as tumor promoter/suppressor, is a dose-dependent process; however, the tumor cell-derived HYALs function mainly as tumor promoter [106].

## 6. Use of HA-Mediated Nanoparticle Systems

CD44 can exist in three different forms: low affinity form, high affinity form induced by inflammation, and constitutive high affinity form. On normal cells CD44 is mostly expressed in low affinity form showing less interactions with HA [115], while cancer cells express constitutive high affinity form of CD44 [116]. This provides rationale to utilize HA-conjugated nanoparticles to target CD44-overexpressed cancer cells [14, 15, 117–119].

The main drawbacks of traditional chemotherapy are severe off-target side effects and unwanted toxicity due to the systemic distribution of the chemotherapeutic drugs [120]. Another major challenge is the development of multidrug resistance (MDR) by the tumor cells, which ultimately makes chemotherapy less effective [121–123]. To overcome these limitations, investigators have developed carrier systems that can selectively deliver cytotoxic doses of drugs to cancer cells avoiding the surrounding normal tissue [124–126].

Recently the use of nanoparticle systems for both diagnostic imaging and drug delivery has attracted increasing attention [127]. Their nanometric dimensions and large surface to volume ratio render them suitable for attaching multiple copies of a variety of ligands. In addition, nanoparticles' unique magnetic, optic, or fluorescent properties make them suitable for biological imaging [128–131]. The nanoparticles used in the biomedical applications include liposomes, polymeric micelles, block ionomer complexes, dendrimers, inorganic and polymeric nanoparticles, nanorods, and quantum dots. All have been tested preclinically or clinically for targeted drug and gene delivery and as agents to enhance dark contrast in magnetic resonance imaging (MRI) [132–134].

Although nanoparticles can be nonspecifically taken up by macrophages, their surface modification with specific ligands to actively target tumor cell specific receptors can potentially enhance the efficiency and selectivity of the delivery [128, 135]. Several studies have indicated that conjugation of HA to a nanocarrier coupled to anticancer drugs such as epirubicin [124], doxorubicin (DOX) [125], paclitaxel (PTX) [126], and mitomycin C (MMC) [124], as well as siRNA, can deliver these agents to CD44-overexpressing cells [136, 137]. The nanoparticles used in these studies have been diverse and include quantum dots [138], carbon nanotubes [139] and nanodots [137, 140], grapheme [141], gold nanoparticles [142], iron oxide nanoparticles [143], and silica nanoparticles. For example, Cho et al. [144] used hyaluronic acid-ceramide- (HA-CE-) based self-assembled nanoparticles for the selective delivery of docetaxel (DET) to the CD44-overexpressing cell line (MCF-7) and concluded that the HA-CE-based nanoparticles might be a good anticancer drug delivery system through passive and active tumor targeting. Eliaz and Szoka [125] demonstrated that doxorubicin (DOX) encapsulated in HA-conjugated liposomes was significantly more potent to CD44-overexpressing cells (B16F10, murine melanoma cell line) compared with free DOX and significantly less toxic than the free DOX to CD44-low expressing cells (CV-1, African green monkey kidney cells). They concluded that liposome encapsulated DOX might be a useful targeted drug carrier for the treatment of CD44-overexpressing cells. Most recently, Shen et al. [145] tested coating solid lipid nanoparticles with hyaluronan (HA-SLNs) for targeted delivery of paclitaxel (PTX) to CD44-overexpressing B16F10 melanoma cells. The* in vitro* results showed that PTX-loaded HA-SLNs led to efficient intracellular delivery of PTX and induced a significant amount of apoptosis in CD44^bright^ cells. During* in vivo* experiments with the B16F10-CD44^bright^ lung metastasis model, PTX-loaded HA-SLNs targeted the tumor-bearing lung tissue well resulting in significant antitumor effects with a comparatively low dose of PTX.

In recent years, HA-mediated iron oxide nanoparticles have also been used for targeted delivery [14, 128, 146–148]. For example, Kumar et al. [143] developed HA-iron oxide (HA-Fe_2_O_3_) nanoparticles and tested their ability to deliver peptides to HEK293 and A549 cells, concluding that HA-Fe_2_O_3_ nanoparticles can be effective tissue and cell targeting systems. More specifically, the use of magnetic nanoparticles such as superparamagnetic iron oxide nanoparticles (SPIONs) functionalized with HA has been investigated for targeting CD44-overexpressed tumor malignancies and inflammations. SPIONs are inorganic particles having an iron oxide core coated by inorganic materials such as silica and gold and organic materials such as phospholipids, fatty acids, polysaccharides, peptides, or other surfactants and polymers [149–151].

In contrast with the nanoparticles previously discussed, SPIONs' inducible magnetic properties facilitate them to be aligned in a defined location in the presence of an externally applied alternating current (AC) magnetic field. This property of inducible magnetism of SPIONs renders them suitable for many biological applications in tumor biology, ranging from diagnostics (MRI) to therapeutics (magnetic hyperthermia), and magnetically assisted transfection of cells [151–154].

In terms of using SPIONs as targeting agent for CD44-overexpressed cells, several investigators used HA-conjugated SPIONs [128, 146–148]. Kamat et al. [128] designed and synthesized dextran coated SPIONs conjugated with HA (HA-DESPIONs) on the surface to target activated CD44-overexpressed macrophages which play a crucial role in atherosclerotic plaque development. They characterized HA-DESPIONs by transmission electron microscopy, thermogravimetric analysis, elemental analysis, dynamic light scattering, and high-resolution magic angle spinning NMR and also verified their biocompatibility and colloidal stability in the presence of serum. They concluded that these nanoparticles can potentially become a useful carrier system for molecular imaging and targeted drug delivery to activated macrophages. El-Dakdouki et al. [146] further elaborated that magnetic nanoparticles conjugated with HA enabled the imaging of atherosclerotic plaques* in vivo* by MRI. They concluded that the very low dose of nanoparticles with high biocompatibility was able to image atherosclerotic plaques without much delay, establishing these nanoparticles as contrast agents for plaque imaging. The same group of the investigators developed DOX loaded HA-coated nanoparticles (DOX-HA-SPIONs) for imaging and drug delivery to cancer cells [14]. Their studies demonstrated that DOX-HA-SPIONs were much more effective than free DOX in damaging not only drug-sensitive but also multidrug-resistant cancer cells which was attributed to the differential uptake mechanisms and cellular distributions of free DOX and DOX-HA-SPIONs in cancer cells.

In addition to using HA-SPIONs as contrast enhancing agents and drug delivery systems, there are several other therapeutic aspects that can be tested using these nanoparticles. Our group has recently tested the cytotoxicity, radiosensitivity, and hyperthermia sensitivity of HA-DESPIONs in CD44 expressing HNSCC cell lines at clinically relevant radiation dose and temperatures, respectively [155]. Our results demonstrated that HA-DESPIONs are nontoxic and although they do not enhance radiation sensitivity, they did increase the effect of local hyperthermia. These results support further development of drug-attached HA-DESPIONs in combination with radiation for targeting cancer stem cells and the development of an alternating magnetic field approach to activate the HA-DESPIONs attached to cancer stem cells.

The latter is an emerging strategy for treating cancers through ablative thermotherapy which may offer patients an alternative and minimally invasive treatment option [156, 157]. Magnetic iron oxide nanoparticle induced hyperthermia is being investigated for the treatment of different cancers using both* in vitro* and* in vivo* mouse xenograft models [156, 158]. Zhao et al. [156] demonstrated that magnetic nanoparticle-based hyperthermia can be achieved by applying an alternating magnetic field. Using a mouse xenograft model of human head and neck cancer (Tu2I2 cell line), they showed that the central tumor temperature was dramatically elevated from room temperature to about 40°C within the first 5–10 minutes which resulted in hyperthermia-mediated cell death due to oncotic necrosis. Huang and Hainfeld [159] reported that magnetic nanoparticles, with a well-tolerated intravenous dose in the presence of applied field of 38 kA/m at 980 kHz, were able to heat up tumors to about 60°C in 2 minutes, while avoiding normal surrounding tissues. Most recently, Thomas et al. [160] developed HA-coated PEGylated SPIONs (HA-PEG-SPIONs) and HA-SPIONs and performed hyperthermia studies using SCC7 cell line (squamous cell carcinoma of head and neck). The* in vitro* results showed 40% reduction in cell viability for both HA-SPIONs and HA-PEG-SPIONs in AMF treated cells. Our preliminary studies using HA-DESPIONs bound to CD44-overexpressing cells in an AMF showed promising data where significant apoptotic cell death was induced in the CD44^bright^ population.

## 7. Conclusion

Despite the recent success of monoclonal antibodies mediated targeting pathways of CD44 variants and improvement in nanoparticle systems for imaging and treatments, there are several unsolved mysteries and lack of knowledge regarding their clinical application in human trials. This paper reviewed the recent scientific literature regarding the role of HA-CD44 signaling pathways, association of different CD44 variants in varieties of tumor types, and four major ways of targeting CD44 receptors for the treatment. The bulk of the evidence indicates that HA-CD44 interaction plays a crucial role in tumor progression and understanding HA-CD44 regulated signaling pathways may lead to early detection and improvement in the treatments. Research suggests targeted elimination of CD44 variant isoforms by the use of monoclonal antibodies against these variants in combination with standard radio/chemotherapy agents may be a promising future treatment for deadly locoregionally advanced malignancies.

## Figures and Tables

**Table 1:** CD44v expression in varieties of tumor types. Table is adapted from Martin et al., 2003 [162].

Human tumors	Change in CD44 expression	Association in tumor progression
Acute myeloid leukemia	2 CD44v6	Correlates with poor prognosis [31]
Colorectal carcinoma	CD44v3	Correlates with poor prognosis [163]
Gastric carcinoma	3 CD44v5, v6, v9	Upregulated during disease progression [162, 164]
HCC	4 Upregulation of CD44s and v5, v6, v7-8, v10	Correlates with poor prognosis [38]
Non-small cell lung carcinomas	Upregulation of CD44v6	Correlates with metastases and poor prognosis [51, 165]
Melanoma	5 CD44v3	Correlates with metastases [37]
Multiple myeloma	CD44v9	Upregulated during disease progression [166]
Nodular sclerosing Hodgkin's disease	6 CD44v10	Upregulated during disease progression [167]
7 Non-Hodgkin's lymphoma	8 CD44v6	Correlates with poor prognosis [168, 169]
Oesophageal squamous cell carcinoma	9 v2	Correlates with poor prognosis [170]
Oral squamous cell carcinoma	Downregulation of CD44v4, v5, v9	Correlates with metastases and poor prognosis [39, 44]
Pancreatic adenocarcinoma	CD44v6	Correlates with poor prognosis [67]
Primary pancreatic cancer	CD44v2 and v6	Correlates with poor prognosis [68]
Thyroid carcinoma	Downregulation of CD44s	Correlates with poor prognosis [171]
Urothelial carcinoma	CD44v6	[172, 173]
Uterine cervical carcinoma	CD44v6, v7-8	Correlates with poor prognosis [174, 175]
